# Comprehensive evaluation of digital village development in the context of rural revitalization: A case study from Jiangxi Province of China

**DOI:** 10.1371/journal.pone.0303847

**Published:** 2024-05-16

**Authors:** Huixin Liu, Yuqian Zhang, Simeng Wang, Hui Zhao

**Affiliations:** 1 School of Marxism, Qingdao University of Technology, Qingdao, Shandong Province, China; 2 School of Management Engineering, Qingdao University of Technology, Qingdao, Shandong Province, China; 3 Simeng Wang, School of Management Engineering, Qingdao University of Technology, Qingdao, Shandong Province, China; 4 Hui Zhao, School of Management Engineering, Qingdao University of Technology, Qingdao, Shandong Province, China; Zhongnan University of Economics and Law, CHINA

## Abstract

Digital rural construction is a key strategic direction to promote China’s rural revitalization and alleviate global climate problems. In order to put forward feasible suggestions for the subsequent development and ensure the smooth development of digital village construction, how to reflect the development level of the digital village through scientific and reasonable comprehensive evaluation has become an urgent problem to be solved. This paper establishes a comprehensive evaluation index system through the Delphi method and principal component analysis method, then assigns weights to the evaluation indicators based on the improved CRITIC-G1 method, and then grades the development level of digital villages according to the extension matter element method. Finally, taking Jiangxi Province in China as an example, the overall development level of digital villages in Jiangxi Province is evaluated from the provincial level according to the proposed method. And put forward the corresponding countermeasures and suggestions. Results: Firstly, the development level of digital villages in Jiangxi Province is good, and there is a trend of excellent development level. Secondly, from different aspects of digital rural development, the digitalization of infrastructure, services, economy, and green production in Jiangxi Province is at a good level, and the digitalization of life has reached an excellent level. Thirdly, from the perspective of development trends, the digitization of infrastructure has a progressive trend towards an excellent level of development, while the digitization of services, economy and green production has signs of development regression. According to the analysis results, the relevant countermeasures and suggestions are put forward from four aspects: talent, capital, governance system and development planning. Other regions can evaluate the development level of the digital village according to the evaluation model proposed in this paper so as to analyze the existing problems and put forward targeted solutions to promote the construction of the digital village.

## 1 Introduction

In the last four decades of analysis, climate change has been one of humanity’s most controversial issues and pressing threats [1]. Since 2020, many countries have proposed carbon-neutral strategies to achieve the goal of global warming below 1.5°C [2]. Agriculture is a significant contributor to global warming, with fertilizer management, intestinal fermentation and rice cultivation all significant sources of greenhouse gases [3]. As the world’s largest country in population and food production, the Chinese government attaches great importance to climate change, actively reduces agricultural greenhouse gas emissions, and makes excellent contributions to climate change mitigation [4]. In 2019, the Chinese government issued the Outline of the Digital Rural Development Strategy, pointing out that it is necessary to develop the rural digital economy vigorously, lead the modernization of agriculture and rural development with digitalization, and realize the digital economy to boost rural revitalization. The deep integration of digital technology and agricultural production can produce an ecosystem with high efficiency, predictability, and adaptability and promote agriculture’s green and low-carbon development [5]. Therefore, the construction of the digital countryside is not only an adequate connection for China’s poverty alleviation and the strategic direction of rural revitalization, but also plays a vital role in solving the global climate problem.

Since China’s digital countryside strategy was first proposed in 2018, construction of the countryside has advanced. By learning the factors that influence the development of the countryside and understanding its current state, one can make workable suggestions for pressing issues that arise during the development process, adaptively alter the course of growth in response to the circumstances, and ensure that digital countryside construction can be developed better and faster. Therefore, it is vital to construct a reasonable evaluation index system and select an appropriate method to fully evaluate the digital countryside’s growth state.

At present, some scholars’ research on digital countryside mainly focuses on interpreting digital countryside development policies and qualitative descriptions of the connotation characteristics of digital countryside construction. Some other scholars look at the development of digital countryside quantitatively and microscopically from a particular perspective, giving macroscopic analysis and empirical tests based on national panel data. Some others study the measurement of digital countryside development level [6–8]. These academic studies provide a certain reference for the scientific and judicious assessment of the development level of the digital countryside, but there are also some things that could be improved. Firstly, the qualitative descriptions are theoretical, lacking in operability and measurability, and weak in practicality. Secondly, the evaluation index screening is selected from a particular perspective, lacking relatively systematic digital rural evaluation indexes, and the coverage is insufficient. The degree of digital countryside development in a region has not yet been thoroughly evaluated by the current study using a rational and scientific assessment system, and this article can fill this research gap.

This article contributes to practice and scholarship as follows. Firstly, using the Delphi method and principal component analysis (PCA), a comprehensive assessment index system of digital rural development is created to address the issue of an incomplete comprehensive assessment index system. Secondly, to address the shortcomings of the current methods for determining the weights of influencing factors in digital village development, the improved CRITIC-G1 method is proposed to be more scientific and reasonable, which establishes a solid basis for the subsequent stage of scientific comprehensive evaluation. Then, the extension matter element model is used to evaluate the rank to which the digital countryside development belongs, which enriches the research of comprehensive evaluation of digital countryside advance and can better reflect the advance level of digital countryside. Finally, combined with the cases, some practical development suggestions are put forward, which provide valuable references for the smooth advance of digital countrysides.

The remainder of this essay is organized as follows. Section 2 introduces the current establishment of digital countryside in various regions, the development model of the digital countryside and the establishment of an evaluation index system. Section 3 constructs a comprehensive assessment index system for digital countrysides growth. Section 4 gives the assignment steps of the improving CRITIC-G1 method and introduces the evaluation steps of applying the extension matter element model. Section 5 takes Jiangxi Province as an example to study the digital village development level from the provincial level. Section 6 discusses the case and gives development suggestions. Conclusions and further work are given in Section 7.

## 2 Review of the literature

**Fig 1:**
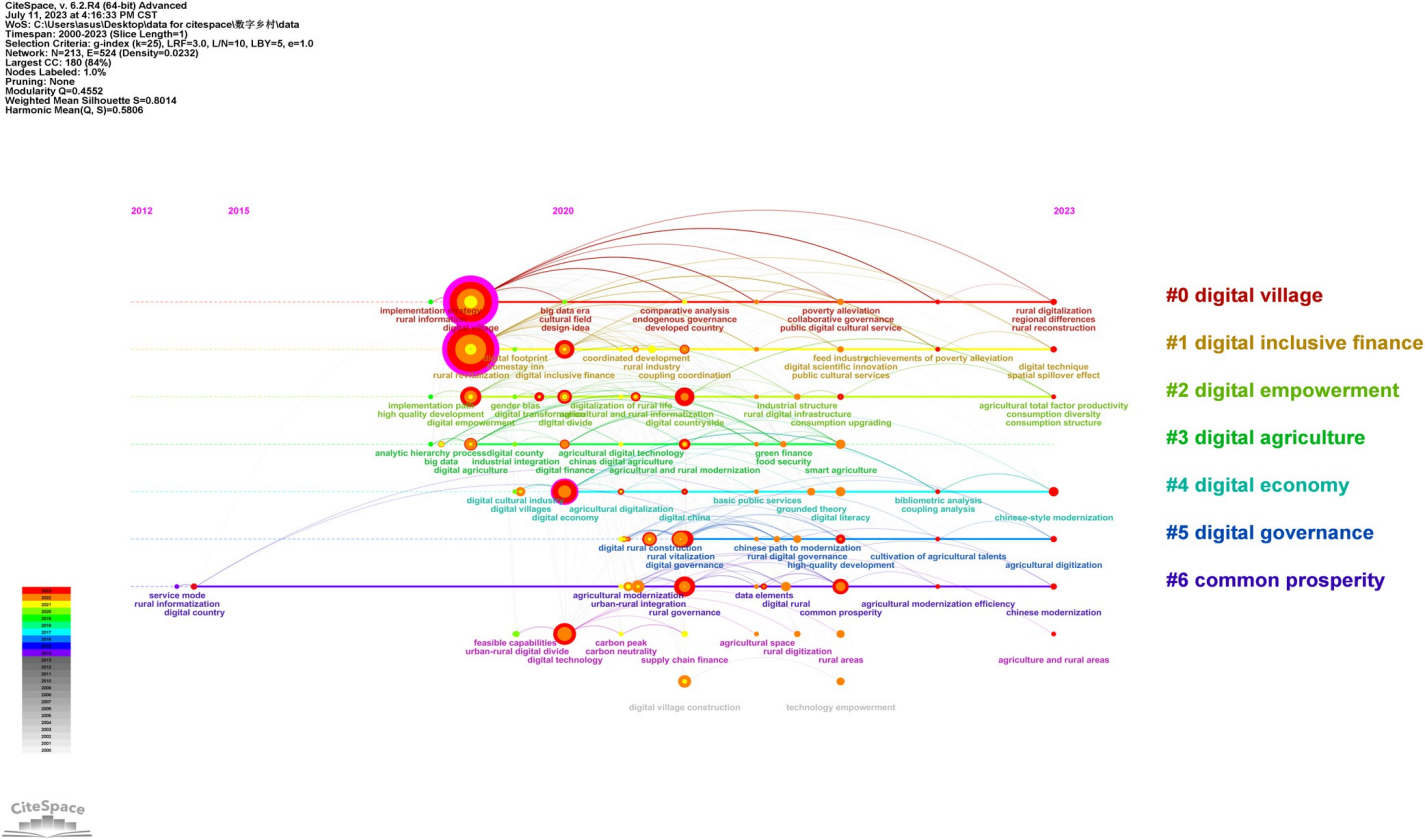
Visual analysis of digital village construction literature.

The digital countryside is an endogenous process for rural advance, along with the use of networking, information technology, and digitization in rural regions, as well as the improvement of farmers’ capacity for using current information technology. The digital countryside has so far drawn attention from all across the world, and many academics have studied it. CiteSpace software was used to analyze the knowledge map of literature related to digital village construction, and the results are presented in Fig 1.

### 2.1 Digital village construction

Some countries and regions have already started digital village construction activities. The Information Network Village Project was started in Korea in 2001 with the intention of bridging the enormous urban-rural digital divide and the rural exodus [9]. In 2014, the Chinese city of Beijing launched a pilot digital village construction project to increase the effectiveness and quality of rural industries, as well as the refinement of rural governance and public services. By the end of 2017, Beijing has basically completed the construction of 135 smart villages, covering 13 districts in the city [10]. In 2015, Hainan Province launched the construction of "Internet Agricultural Towns" with the town as the unit. The "1 + 2 + N" operation model was built with "Internet" as its characteristic [11]. In an effort to transition from a conventional society to one that is digitally empowered and knowledge-based, India began the Digital India Programme in 2015 [12]. In 2016, Japan proposed "Next generation agricultural, forestry and water industry Technology based on Intelligent machinery + Intelligent IT project" to apply digital technology to natural resource management and disaster prevention [13]. The EU introduced the Smart Villages campaign in 2017 and began a two-and-a-half-year Smart Villages 21 project in 2019 to pick 21 countrysides to provide monetary and technical help and ultimately scale up experience of building smart villages in 21 EU villages [14]. Under the framework of the Smart Village movement, Italy, France, Poland, and other countries have launched different digital village construction plans according to their conditions.

At present, the theoretical research and practical application of digital countryside are primarily concentrated in developed countries and regions, such as the United States, the European Union [15], Australia [16], Ireland [17], and Poland [18], while little attention is paid to the rural digitalization policies or planning plans in developing countries [19]. At the same time, it can be seen that the core of national digital rural establishment is not identical. India focuses on digital technology infrastructure construction and improvement. European nations primarily improve the environment for rural development by applying new technologies and strengthening public services [20]. The construction of China’s digital villages is more difficult due to environmental differences between China and other nations, as well as the striking differences between regions within China. However, there has been little in-depth exploration of the content and establishment logic of digital villages as well as no in-depth analysis of the practical cases of China’s digital villages.

### 2.2 Digital village development and evaluation

In the development model of the digital countryside, numerous scholars have conducted research. Arabatzis et al. used the PROMETHEE-II method to evaluate local group actions in implementing LEADER+CI in Greece [21]. Ella et al. used a qualitative research approach to construct a development model for Indonesia that can develop smart villages through an ecosystem approach with a bottom-up planning process as the primary method and a collaborative governance model as the core model [22]. Manasijević et al. construct a development model for the digital transformation of villages that contributes to the relativization of regional disparities in the Republic of Serbia by investigating the advance of digital transformation of Vrmdža village with the Internet and modern digital technologies for engaging in business activities in villages as the core model [23]. Murty et al. take an endogenous approach to the construction of a new development framework for smart villages and divide it into four stages: discovery, planning, outsourcing, and implementation, laying the groundwork for the development of a scalable smart village framework [24]. Kyriakopoulos et al. conducted a comprehensive analysis of the impact of climate change on Greek agriculture through the new agricultural policy, and proposed corresponding critical interventions and actions [25].

Meanwhile, in terms of assessment index system construction, Shen et al. constructed an assessment index system from five aspects from the perspective of agricultural informatization and used the hierarchical analysis method (AHP) to evaluate the degree of agricultural digital development [26]. Based on the connotation of the concept of the digital countryside, Chang et al. constructed an assessment index system for the digital countryside, which contains nine first-level indicators of capability and effectiveness categories, as a way to scientifically evaluate the advanced level of the digital village [27]. Cui et al. explored the theory and designed the main indicators of the rural digital economy around the digital economy composition of industrial digitization and digital industrialization [28]. Zhang et al. measured and evaluated the readiness of China’s provincial digital countryside development by constructing a digital countryside’s advance readiness index system using the AHP method and entropy value method[29]. Mu et al. created an index system of the digital economy in agriculture and rural areas from the industrial level on the basis of three components of digital economy formation [30]. Li Weiwei et al. combined the Boston Consulting Group Matrix and GeoDetector to study the construction level and evolution pattern of digital villages in 70 counties in Guangxi [31]. Zhang Ping et al. used geographic information systems (GIS) to evaluate the development of the digital countryside in Gansu Province and then formulated targeted development strategies according to the development level of each region [32]. Yang Xiaojuan et al. used the AHP and BCG matrix to evaluate the development of rural revitalization in 131 cities in western China and studied rural revitalisation performance’s evolution model and driving mechanism [33].

To sum up, the study of digital countryside has attracted more and more attention. It has made good progress in various fields, providing valuable experience for this paper’s study. However, there are some limitations to the existing research. Firstly, scholars mainly study digital countryside at the theoretical level, focusing on the digital countryside’s connotation, operation mechanism and governance strategy. However, little attention has been paid to the ongoing development of digital villages around the country, and there are no accurate and consistent measurement tools to measure the current development of digital villages correctly. Secondly, most existing studies construct the index system from a specific perspective, such as agricultural informatization, digital economy and industry level, and the coverage of digital rural indicators is insufficient. Factors like farmers’ life services and the rural ecological environment are often ignored. Finally, in terms of evaluation methods, single weighting methods such as analytic hierarchy process (AHP) or entropy value method are generally used. There are biases in subjective judgment or quality biases in objective data, leading to unsatisfactory evaluation results. Therefore, this paper comprehensively considers all aspects of building a sound evaluation index system, including infrastructure, service, economy, life and digital green production. Taking Jiangxi Province as a case, the paper comprehensively evaluates the development of the digital countryside from the provincial level. Then, the CRITIC-G1 weighting method is adopted, which reflects the internal relationship between indicators and the objective characteristics of data. Finally, according to the evaluation results, the development of digital villages in Jiangxi Province is studied and analyzed, and targeted measures are formulated to promote the construction of digital villages. This paper hopes to establish a scientific and reasonable evaluation model to truthfully evaluate the development level of digital villages in various regions and then solve and promote the construction of digital villages according to the reflected problems.

## 3 Construction of a comprehensive evaluation index system for digital village development

The evaluation index system substantially affects the evaluation process, because it is the basis of the whole process and directly affects the accuracy and efficiency of the outcome. Therefore, in the process of construction, influencing factors should be considered from as many perspectives as possible. In order to accomplish the goal, relevant experts were invited, and Delphi and principal component analysis, a combination of qualitative and quantitative methods, were used to give full play to the role of professionals while reducing the correlation between indicators. As a result, we are able to create an index system of comprehensive evaluation for digital village development that is more rational and scientific.

**Fig 2:**
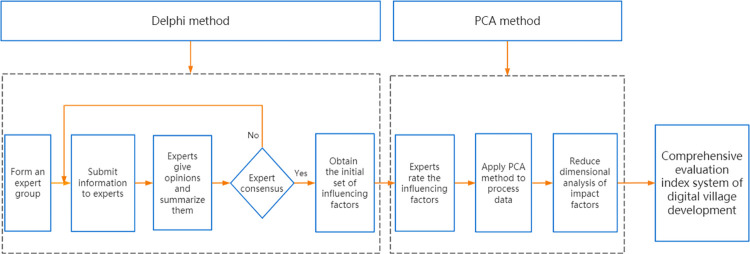
The construction process of the comprehensive evaluation index system of digital village development.

Firstly, the initial set of influencing factors of digital village development was obtained by using the Delphi method for research and analysis. Then the data was processed and analyzed by using the PCA method. Finally, 21 influencing factors were selected and divided into five aspects: infrastructure digitization, service digitization, economic digitization, life digitization and green production digitization, to obtain a complete assessment indicator system of the growth of the digital village, which is presented in Table 1, and the construction process is shown in Fig 2.

**Table 1:** Comprehensive evaluation index system of digital village development.

Primary evaluation index	Secondary evaluation indicators	Explanation of indicators
C1: Infrastructure digitization	D1:Rural broadband access users	The number of subscribers with broadband access per 10,000 rural households [29]
	D2:Rural power generation equipment capacity	The total capacity of various rural power generation facilities [34]
	D3:Rural residents’ costs on transportation and communication	The sum of rural residents’ expenditures on travel and communication [34]
	D4:Rural road mileage	The total number of miles of roads in rural areas [35]
	D5:Rural piped water penetration rate	The ratio of the number of rural persons using piped water to the total rural population [34]
C2: Service digitization	D6: Amount of village committee	The amount of total rural village committees [29]
	D7:Amount of village clinics	The total number of village medical institutions [36]
	D8:Amount of rural social pension insurance participants	The number of people participating in social pension insurance in rural areas [37]
	D9:Amount of rural residents with minimum living allowances	The number of persons with minimum subsistence security in the countryside [35]
C3:Economic digitization	D10:Total length of postal rural delivery routes	The total distance covered by rural postal delivery routes [26]
	D11:Disposable income per capita of rural residents	The proportion of rural people’s total disposable income to their entire population [36]
	D12:Digital Economy Index	The index is used to gauge how far the digital economy has come [38]
	D13:Digital Inclusive Finance Index	Digital financial inclusion morning microdata [39]
C4: Life digitization	D14:Rural residents’ cell phone ownership	The average number of cell phones ownership per 100 rural families [35]
	D15:Computer ownership among rural residents	The average number of computers ownership per 100 rural families [26]
	D16:Population coverage of rural TV programs	The percentage of rural citizens who have access to television to the total population of rural residents [40]
C5: Green production digitization	D17:Effective irrigated area	The area of cultivated land that has been equipped with irrigation works or equipment for regular irrigation in the current year [40]
	D18:Total power of agricultural mechanization	The sum of the power of various power machines in agriculture [41]
	D19:Actual area under machine cultivation in that year	The actual arable land cultivated by agricultural machinery in that year [40]
	D20:Fertilizer application of agricultural fertilizer discounted amount	The actual amount of fertilizer used in agricultural production in terms of discounted amounts [36]
	D21:Comprehensive soil erosion control area	The total area of soil erosion treated by various measures [40]

Among them, the experts selected for this paper must meet one of the following conditions: company personnel with more than five years of practical experience in digital village construction projects; professors from well-known universities with more than three years of research experience in digital villages; government department officials related to digital village construction; lawyers from famous law firms providing national legal advice for digital village projects.

## 4 Research methodology

**Fig 3:**
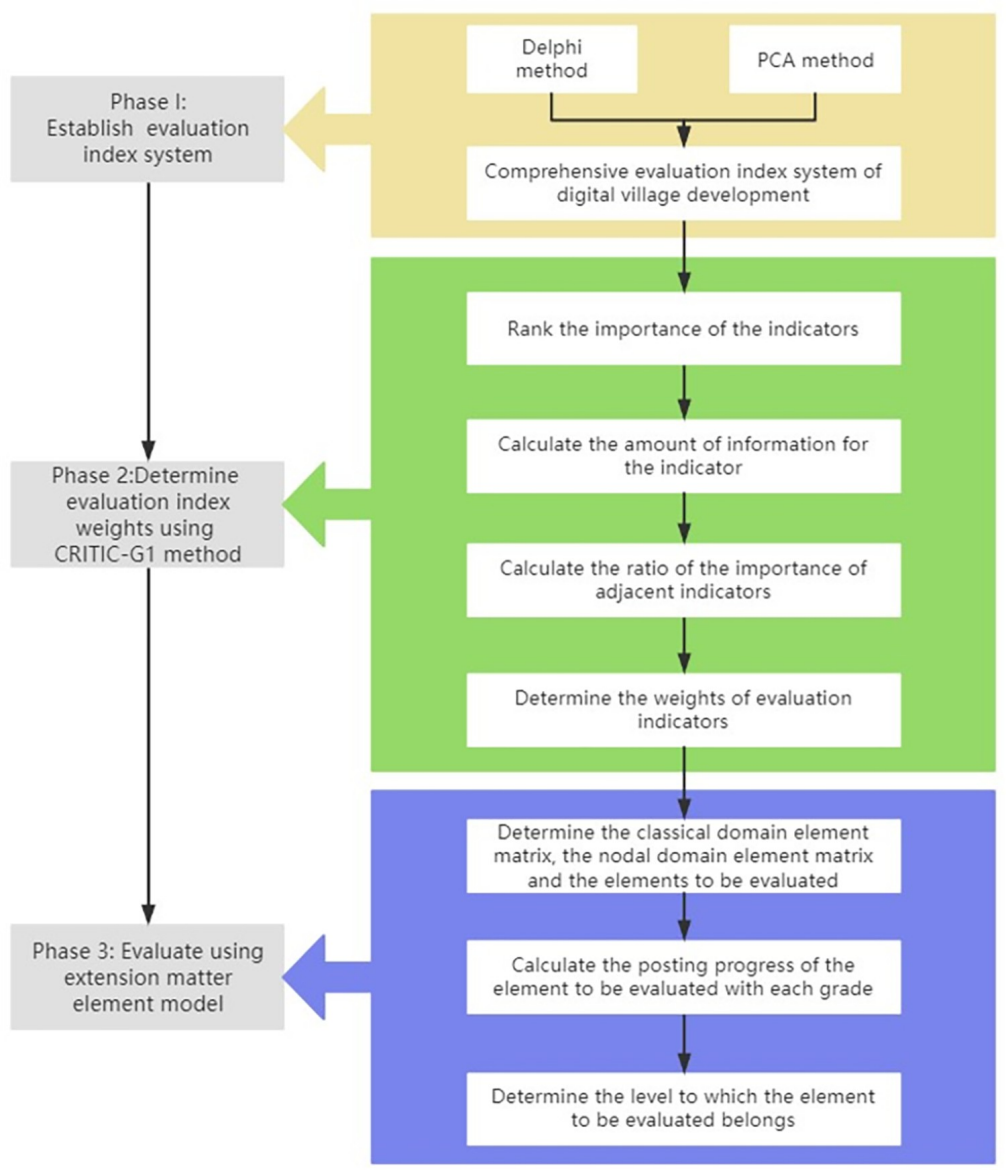
Framework flow chart.

In order to ensure that the study is more scientific and reasonable, this paper uses a combination of the Delphi method and PCA method to select critical factors of digital village development and establish an evaluation index system (see Section 3). Then use the assignment method of improved CRITIC modified G1 to assign total weights to the indicators. Then the evaluation model is established to assess the advance of the digital village by combining with the extension matter-element theory. Finally, some suggestions are given according to the case results. The research framework is displayed in Fig 3.

### 4.1 Improved CRITIC correction G1

At present, many mature methods have emerged for determining the weights of various indicators, such as the AHP method and Delphi method, which rely on experts’ subjective experience, and the weights obtained by these methods are affected by experts’ experience and professionalism. In contrast, objective weighting methods such as the entropy value method, rough set method and standard deviation method that rely on mining objective data information can only reflect objective information and cannot incorporate decision makers’ subjective consciousness into decision-making, which is easily detached from reality. Furthermore, the factors that influence the growth of digital villages are complex and difficult to quantify [29]. Purely subjective or objective empowerment will have an impact on the subsequent comprehensive evaluation, while the comprehensive assignment method combines the benefits of subjective and objective assignment methods, which can reflect the personal will of experts and apply objective theories and methods in the meantime. Therefore, this article selects the comprehensive assignment method that combines subjective and objective.

The G1 weighting method is a modified method of the AHP method, in which experts assign values to ranked indicators based on their importance for a two-by-two comparison and then apply the importance formula to calculate indicator weights [42]. The G1 method can fully reflect the subjective consciousness of the evaluator and find out the intrinsic connection of the importance of the influencing factors based on the sequential relationship between the influencing factors, without testing for consistency. It both ensures the authenticity of subjective judgment and enhances the simplicity of operation. However, this weighting method, which relies on experts’ subjective ratings, is more influenced by experts’ preferences and cannot reflect the changes in objective conditions.

The CRITIC method is an objective weight assignment method, which is proposed by Diakoulaki. Its main goal is to establish the indicators’ objective weights using two fundamental ideas. The first is contrast intensity, which reflects, in terms of standard deviation, the size of the difference between the values obtained by each assessment scheme for the same index. The size of standardized deviation indicates the size of the gap between values taken by schemes within the same indicator, and the more standardized deviation, the more significant the difference in values taken by the various schemes. The second is the conflict between the evaluation index based on the correlation. If two indexes have a significant positive correlation, their conflict is low. The CRITIC method also uses the coefficient of variation to make the dispersion reflected by the standard deviation more relevant to reality on the basis of considering the amount of information of the indicators and factor correlation, which has significant superiority [43]文献32. In contrast, other objective weight assessment methods, such as the entropy weighting method and the TOPSIS method, without considering the interaction and interdependence between indicators, can thus not deal with the multi-index information aggregation assessment.

Therefore, this paper uses the procedure of calculating the information quantity of indicators in the improved CRITIC theory to replace the process of G1 method indicator assignment, and uses the information quantity of indicators as the indicator assignment to construct a comprehensive assignment method of the improved CRITIC-G1 method, which not only retains the importance ranking of indicators but also reflects the size of the information quantity contained in indicators, making the assignment results more objective and reasonable [44]. The precise steps of the calculation are listed below.

Step 1. Importance ranking. Five professionals with expertise in the digital village were invited to rank the importance level of evaluation indicator *i* based on their own experience in the field.

The improved CRITIC method determines the objective weight of indicator *i* by calculating the product of the contrasting intensity within the evaluation indicator *i* itself and the conflicting product between each other indicator. If the more information content of indicator *i* has, the more information it has and transmits, the greater the impact on the evaluation object and the greater the weight.

Step 2. Determine the comparison intensity of the evaluation index *i* itself *c*_*v*(*i*)_, dividing the standard deviation of indicator *i* by its mean value.

$$c_{v(i)}=\sigma_{\left(i\right)}/\mu_{\left(i\right)}$$ (1)

Where *σ*_(*i*)_ is the standard deviation of the *i* th assessment index; *μ*_(*i*)_ is the mean value of the *i* th assessment index.

Step 3. Calculate the conflict between evaluation indicators Cf(*i*).

$$Cf(i)=\sum_{k=1}^{i}\;(1-r_{ki})$$ (2)

Where *r*_*ki*_ is the correlation coefficient between assessment index *k* and *i*.

Step 4. Calculate the amount of information C_*i*_.


$$C_{i}=c_{v(i)}*Cf(i)$$ (3)

Step 5. Calculate the ratio of importance degree *r*_*i*_. Determine the importance ratio of adjacent indicators by calculating the information content of indicators.


$$r_{i}=\{\begin{array}{l} c_{i}/c_{i+1},\mathrm{if}\;c_{i}>c_{i+1}\\ 1,\;\mathrm{if}\;c_{i}<c_{i+1}\\ -,\;\mathrm{if}\;c_{i+1}\;\mathrm{does}\;\mathrm{not}\;\mathrm{exist} \end{array}$$ (4)

Step 6. Determine the evaluation index weights. According to the ratio of importance *r*_*i*_,*r*_*i*−1_,….*r*_1_, the weight of the *i* th indicator to the assessment object is obtained.

$$w_{i}={\left(1+\sum_{k=1}^{i-1}\prod_{m=k}^{i-1}r_{m}\right)}^{-1}$$ (5)

Where *i* is the total number of tertiary indicators under a secondary indicator.

From the weight w_*i*_, the weights of the indicators *i*-1,*i*-2,…,1 can be obtained.


$$w_{k-1}=r_{k-1}\;*\;w_{k},\;k=i,i‐1,\ldots ,2$$ (6)

### 4.2 Extension matter element model

Topology is the law and method to study the possibility of expanding things and to explore and innovate, which is applied to solve contradictory problems. The fundamental goal of matter element analysis is to use ordered triples to represent the three components of things: things, characteristics as well as magnitude, and this triplet is called matter element. Topology element evaluation focuses on evaluating the feasibility and optimality of the solution. Based on the topology theory, the evaluation scheme is taken as the object element, and the evaluation criteria are determined through the qualitative analysis of the topology of the object element, and then the correlation function is introduced for the quantitative calculation to get the affiliation degree of each evaluation scheme to the evaluation criteria, which combines the qualitative and quantitative analysis and takes into account the compatibility of the evaluation indexes. The consequences can be measured more scientifically and rationally [45]. The main steps are as follows.

Step 1. Determine the classical domain element matrix.

$$R_{j}=(Q_{j},V_{i},C_{ij})=\left[\begin{array}{ccc} Q_{j} & v_{1} & c_{1j}\\ & v_{2} & c_{2j}\\ & . & .\\ & . & .\\ & . & .\\ & v_{n} & c_{nj} \end{array}\right]=\left[\begin{array}{ccc} Q_{j} & v_{1} & \left(a_{1j},b_{1j}\right)\\ & v_{2} & \left(a_{2j},b_{2j}\right)\\ & . & .\\ & . & .\\ & . & .\\ & v_{n} & \left(a_{nj},b_{nj}\right) \end{array}\right]$$ (7)

Where the digital village development is divided into j assessment levels, denoted by *Q*_*j*_, the classification of levels depends on the specific situation; the set of indicators is denoted by V (*v*_1_, *v*_2_,…,*v*_*n*_), including a total of n indicators; the classical domain *C*_*ij*_ is the range of data of the assessed actual values of the indicator set *v*_*i*_ under different assessment levels *Q*_*j*_.

Step 2. Determine the nodal domain element matrix.

$$R_{Q}=(Q,V_{i},C_{ip})=\left[\begin{array}{ccc} Q & v_{1} & c_{1p}\\ & v_{2} & c_{2p}\\ & . & .\\ & . & .\\ & . & .\\ & v_{n} & c_{np} \end{array}\right]=\left[\begin{array}{ccc} Q & v_{1} & \left(a_{1q},b_{1q}\right)\\ & v_{2} & \left(a_{2q},b_{2q}\right)\\ & . & .\\ & . & .\\ & . & .\\ & v_{n} & \left(a_{nq},b_{nq}\right) \end{array}\right]$$ (8)

Where the nodal domain *C*_*ip*_ is the range of data of the assessed real values of the assessment indicator set V under the whole assessment level Q.

Step 3. Determine the elements to be evaluated.

$$R_{0}=(Q_{0},V_{i},C_{i})=\left[\begin{array}{ccc} Q_{0} & v_{1} & c_{1}\\ & v_{2} & c_{2}\\ & . & .\\ & . & .\\ & . & .\\ & v_{n} & c_{n} \end{array}\right]$$ (9)

Where C (*c*_1_, *c*_2_,…,*c*_*n*_) denotes the evaluation data values of the elements to be evaluated under different indicators.

Step 4. Calculate the posting progress of the element to be evaluated with each grade *K*_*j*_(*R*_0_).

$$D_{j}(v_{i})=|v_{i}-\frac{a_{ij+b_{ij}}}{2}|-\frac{1}{2}(b_{ij}-a_{ij})$$ (10)

$$K_{j}(R_{0})=1‐\frac{1}{n(n+1)}\sum_{i=1}^{n}D_{j}(v_{i})w_{i}(X)$$ (11)

Where *D*_*j*_(*v*_*i*_) and *w*_*i*_(*X*) refer to the separation of the element to be assessed from the classical domain and the size of the indicator weight, respectively.

Step 5. Determine the class to which it belongs. If there is material element posting progress to be evaluated *K*_*j*’_(*Q*_0_) = max{*K*_*j*_(*Q*_0_)} (j = 1,2,3,4), then it can be determined that the element to be evaluated *R*_0_ belongs to class *j*’.

## 5 Case study

### 5.1 Case description

Jiangxi Province is made up of eleven prefecture-level cities, situated in the middle and lower portions of the Yangtze River in southeast China. It has a prime position and easy access to transportation. Jiangxi Province released "Opinions on the Implementation of Digital Countryside Development Strategy in Jiangxi Province " in 2020 to fully implement the digital countryside development strategy, increase the application of informatization in rural regeneration to its fullest potential and drive the modernization of agriculture and rural areas as a whole, carrying out the spirit of the "Digital Countryside Development Strategy Outline". The "Three-Year Action Plan for the Construction of Digital Agriculture and Rural Areas in Jiangxi Province" was released by Jiangxi Province in 2021. It outlined plans for the establishment of the digital countryside from 2021 to 2023 and created a number of objectives and actions to raise the level of digital growth in Jiangxi Province’s rural districts. It is thus evident that Jiangxi Province maintains a high priority on the digital rural strategy. Jiangxi Province’s digital countryside development level is 42.0%, depending on the "China Digital Countryside Development Report" published in 2022, which surpasses the national average and is located in eighth place, serving as a proxy for China’s overall state of digital rural development. Therefore, this article takes Jiangxi Province of China as an example and collects relevant data for the last six years, from 2016 to 2021, to make a comprehensive evaluation of digital countryside advance in Jiangxi Province from the provincial level. The China Statistical Yearbook and Jiangxi Provincial Statistical Yearbook are the primary sources of research data in this article. The interpolation method is used to fill in for the issue of some specific indicators having missing content in some years.

### 5.2 CRITIC-G1 empowerment and evaluation of topological elements

**Fig 4:**
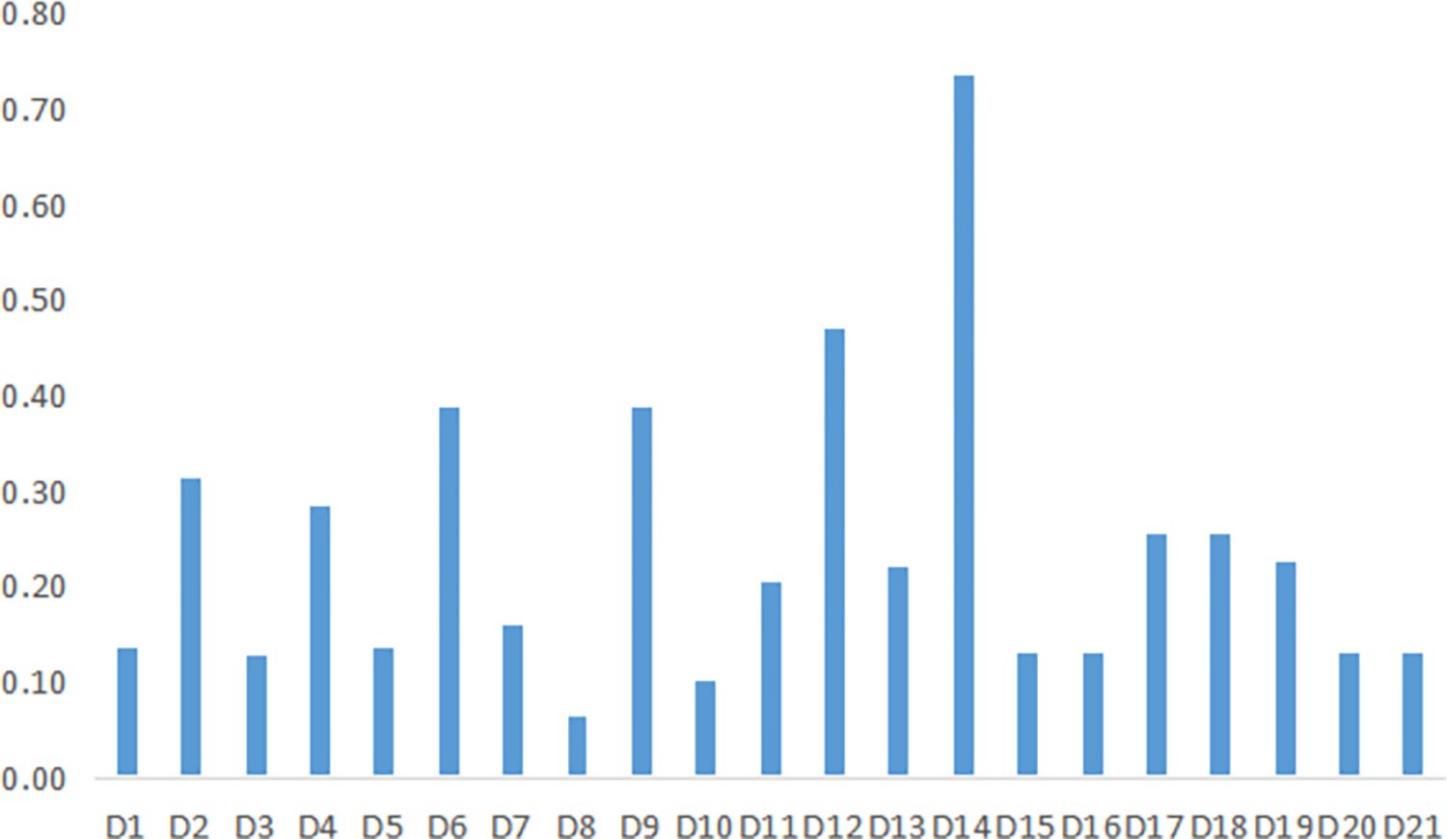
Indicator weighting chart.

Step 1. Determine the index weights. Depending on the digital countryside development index system (see Table 1), the index weights are determined by the method of improving CRITIC correction G1, and the index system weights of this paper are shown in Table 2, as shown in Fig 4.

**Table 2:** Calculation results of indicator weights.

Guideline layer	Indicator layer	Contrast intensity	Conflict	Amount of information	Importance ratio	Indicator weights
C1:Infrastructure digitization	D2:Rural power generation equipment capacity	0.3938	2.9788	1.1732	1.1101	0.3150
	D4:Rural road mileage	0.1490	7.0910	1.0568	2.0750	0.2838
	D5:Rural piped water penetration rate	0.0706	7.2132	0.5093	1.0000	0.1368
	D1:Rural broadband access users	0.2648	7.2794	1.9273	1.0713	0.1368
	D3:Rural residents’ costs on transportation and communication	0.2537	7.0927	1.7991	**—**	0.1277
C2:Service digitization	D6:Amount of village committees	0.0013	4.9864	0.0067	1.0000	0.3877
	D9:Amount of rural residents with minimum living allowances	0.0991	5.0205	0.4978	2.4107	0.3877
	D7:Amount of village clinics	0.0409	5.0516	0.2065	2.5183	0.1608
	D8:Amount of rural social pension insurance participants	0.0502	1.6333	0.0820	**—**	0.0639
C3: Economic digitization	D12:Digital Economy Index	0.3330	6.0192	2.0046	2.1341	0.4711
	D13:Digital Inclusive Finance Index	0.1546	6.0771	0.9393	1.0737	0.2208
	D11:Disposable income per capita of rural residents	0.1455	6.0120	0.8749	2.0059	0.2056
	D10:Total length of postal rural delivery routes	0.1045	4.1737	0.4362	**—**	0.1025
C4: Life digitization	D14:Rural residents’ cell phone ownership	0.0680	4.2872	0.2917	5.5988	0.7368
	D16:Population coverage of rural TV programs	0.0118	4.4150	0.0521	1.0000	0.1316
	D15:Computer ownership among rural residents	0.0563	4.2420	0.2390	**—**	0.1316
C5: Green production digitization	D17:Effective irrigated area	0.0012	5.6350	0.0068	1.0000	0.2557
	D18:Total power of agricultural mechanization	0.0682	6.2484	0.4258	1.1276	0.2557
	D19:Actual area under machine cultivation in that year	0.0603	6.2636	0.3776	1.7329	0.2268
	D20:Fertilizer application of agricultural fertilizer discounted amount	0.1037	2.1012	0.2179	1.0000	0.1309
	D21:Comprehensive soil erosion control area	0.0380	6.1743	0.2348	**—**	0.1309

It can be seen that the weight of D2 rural power generation equipment capacity, D4 rural road mileage, D6 number of village committees, D9 minimum number of rural residents living security, D12 digital economy index and D14 rural residents mobile phone ownership is significant, indicating that they are the main factors affecting the evaluation of digital rural development. At present, the rural infrastructure construction in Jiangxi Province is relatively weak, and the living conditions of villagers are not concerned enough, so these indicators are the key factors to consider when evaluating. The digital economy can enable rural industries to achieve digital transformation, improve production efficiency and quality, and increase farmers’ income, which plays a vital role in developing the digital countryside. Secondly, D8 the number of participants in rural social endowment insurance and D10 the total length of postal delivery routes in rural areas have less weight. In recent years, Jiangxi Province attaches great importance to the issue of residents’ pension insurance and implements the national insurance program, so the issue of rural residents’ pension insurance participation has achieved positive results, resulting in a substantial increase in the number of participants. Due to the rapid rise of e-commerce, the limitations of traditional business models have been broken so that rural areas can enjoy the same convenient shopping life as urban areas. Therefore, the impact of the two indicators on the development of the digital countryside is relatively tiny.

Step 2. Classify the evaluation levels. According to the relevant literature, after whole discussion by experts, it was determined that the evaluation level of digital countryside advance was divided into four levels, with level Ⅰ indicating "unqualified", level Ⅱ indicating "qualified", level Ⅲ indicating "good " and level IV indicating "excellent". The corresponding threshold values are displayed in Table 3.’

**Table 3:** Development-level assessment criteria.

Grade	I	II	III	Ⅳ	Nodal Domain
Rating	(0.0, 2.5)	(2.5, 5.0)	(5.0, 7.5)	(7.5, 10.0)	(0, 10.0)

Step 3. On the basis of the criteria in Table 3, determine the classical domain matrix *R*_*j*_, the nodal domain matrix *R*_*Q*_ and the elements to be evaluated *R*_0_ respectively by Eqs (7)–(9).


$$R_{j}=(Q_{j},V_{i},C_{ij})=\left[\begin{array}{cccccc} Q_{j} & V_{i} & j=Ⅰ & j=Ⅱ & j=Ⅲ & j=Ⅳ\\ & v_{1} & \left(0,2.5\right) & \left(2.5,5.0\right) & \left(5.0,7.5\right) & \left(7.5,10.0\right)\\ & v_{2} & \left(0,2.5\right) & \left(2.5,5.0\right) & \left(5.0,7.5\right) & \left(7.5,10.0\right)\\ & . & . & . & . & .\\ & . & . & . & . & .\\ & . & . & . & . & .\\ & v_{21} & \left(0,2.5\right) & \left(2.5,5.0\right) & \left(5.0,7.5\right) & \left(7.5,10.0\right) \end{array}\right]$$

$$R_{Q}=(Q,V_{i},C_{ip})=\left[\begin{array}{ccc} Q & v_{1} & \left(0,10\right)\\ & v_{2} & \left(0,10\right)\\ & . & .\\ & . & .\\ & . & .\\ & v_{21} & \left(0,10\right) \end{array}\right]$$

$$R_{0}=(Q_{0},V_{i},C_{i})=\left[\begin{array}{ccc} Q_{0} & v_{1} & 6.3\\ & v_{2} & 7.0\\ & v_{3} & 6.6\\ & v_{4} & 6.8\\ & v_{5} & 7.7\\ & v_{6} & 7.2\\ & v_{7} & 6.3\\ & v_{8} & 4.6\\ & v_{9} & 5.1\\ & v_{10} & 7.8\\ & v_{11} & 6.4\\ & v_{12} & 5.7\\ & v_{13} & 4.8\\ & v_{14} & 8.2\\ & v_{15} & 5.2\\ & v_{16} & 8.5\\ & v_{17} & 6.2\\ & v_{18} & 6.3\\ & v_{19} & 5.8\\ & v_{20} & 5.1\\ & v_{21} & 4.7 \end{array}\right]$$

Step 4. Determine the posting progress. From the Formula (10), the distance of 21 indicators about four levels of Ⅰ, Ⅱ, Ⅲ, and Ⅳ can be calculated, and the results are displayed in Table 4.

**Table 4:** Distance of indicators on the four assessment levels.

Indicators	Grade I	Grade II	Grade III	Level IV
*v*_1_	3.8	1.3	-1.2	1.2
*v*_2_	4.5	2.0	-0.5	0.5
*v*_3_	4.1	1.6	-0.9	0.9
*v*_4_	4.3	1.8	-0.7	0.7
*v*_5_	5.2	2.7	0.2	-0.2
*v*_6_	4.7	2.2	-0.3	0.3
*v*_7_	3.8	1.3	-1.2	1.2
*v*_8_	2.1	-0.4	0.4	2.9
*v*_9_	2.6	0.1	-0.1	2.4
*v*_10_	5.3	2.8	0.3	-0.3
*v*_11_	3.9	1.4	-1.1	1.1
*v*_12_	3.2	0.7	-0.7	1.8
*v*_13_	2.3	-0.2	0.2	2.7
*v*_14_	5.7	3.2	0.7	-0.7
*v*_15_	2.7	0.2	-0.2	2.3
*v*_16_	6.0	3.5	1.0	-1.0
*v*_17_	3.7	1.2	-1.2	1.3
*v*_18_	3.8	1.3	-1.2	1.2
*v*_19_	3.3	0.8	-0.8	1.7
*v*_20_	2.6	0.1	-0.1	2.4
*v*_21_	2.2	-0.3	0.3	2.8

Step 5. Determine the assessment levels. The development assessment of each aspect can be calculated from the Formula (11) and the data in Table 4 about the posting progress of different levels as well as the comprehensive posting progress, so as to determine the evaluation level of each aspect as well as the comprehensive evaluation level, as presented in Table 5.

**Table 5:** Correlation and evaluation grade of various aspects of digital village development in Jiangxi Province.

Different aspects	Grade I	Class II	Grade III	Level IV	Evaluation Level
Infrastructure digitization	0.8536	0.9369	1.0203	0.9797	Grade III
Services digitization	0.8212	0.9462	1.0161	0.9287	Grade III
Economic digitization	0.8320	0.9570	1.0241	0.9180	Grade III
Life digitization	0.5546	0.7692	0.9482	1.0287	Level IV
Green production digitization	0.8902	0.9735	1.0256	0.9432	Grade III
Comprehensive posting progress	0.9568	0.9838	1.0034	0.9891	Grade III

As seen in Table 5, the comprehensive development evaluation of the digital village in Jiangxi Province is at a good level. Since *K*_IV_>*K*_II_, it indicates that the digital village in Jiangxi Province is developing in an excellent direction and has an increasing development trend. From different aspects, the digitalization of infrastructure, services, economy, and green production is well developed, and the digitalization of life is at an excellent level, which is more prominent. At the same time, infrastructure is developing in an excellent direction. However, in the digitization of services, economy, and green production, there is a trend towards a qualified direction, which is a downward trend. Therefore, it is necessary to identify the reasons for the downward trend in the digitization of services, economy and green production and then formulate targeted measures to strengthen their development. Then, the government continues to grasp the current strong momentum of digital development of infrastructure and life to improve the overall development level of the digital countryside in Jiangxi Province.

### 5.3 Sensitivity analysis

**Fig 5:**
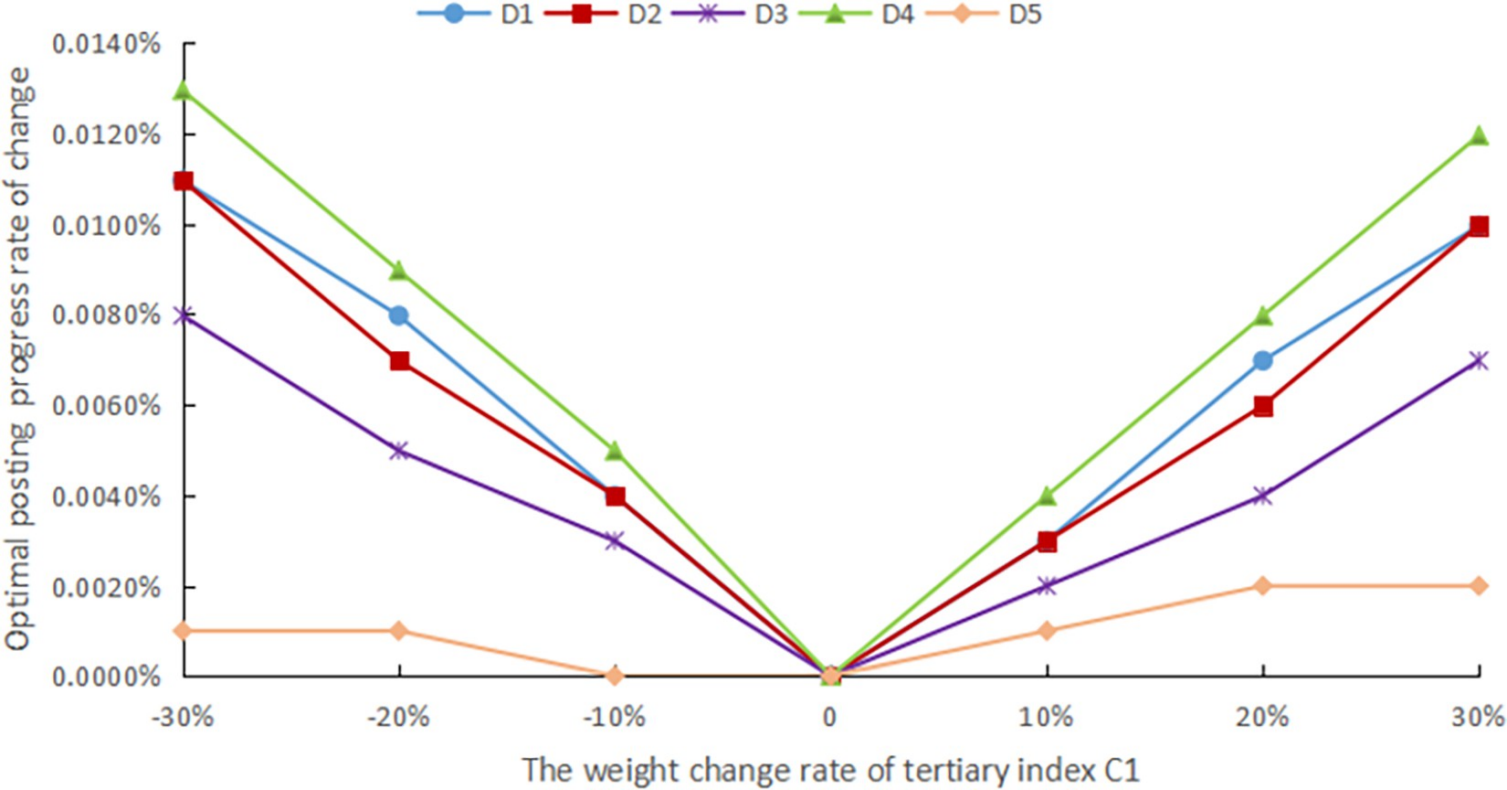
The weight change rate of tertiary index C1.

**Fig 6:**
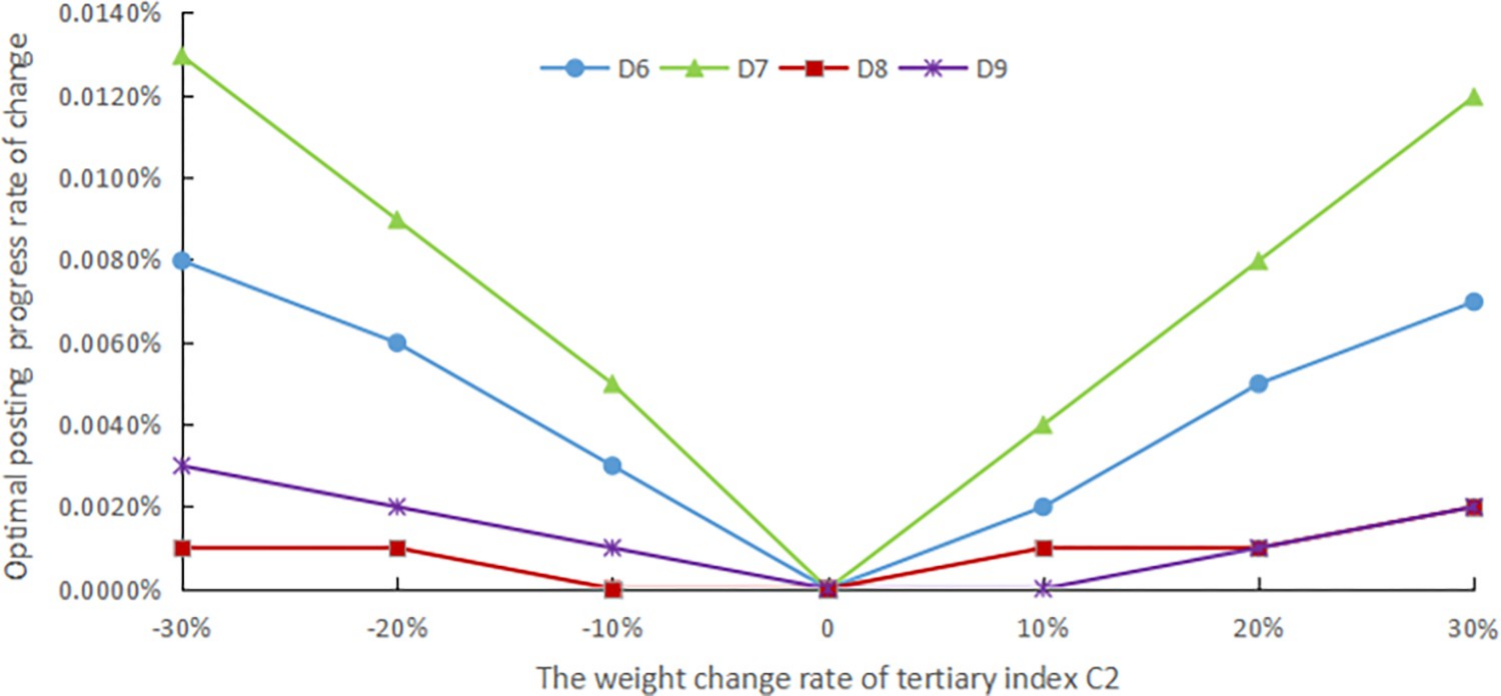
The weight change rate of tertiary index C2.

**Fig 7:**
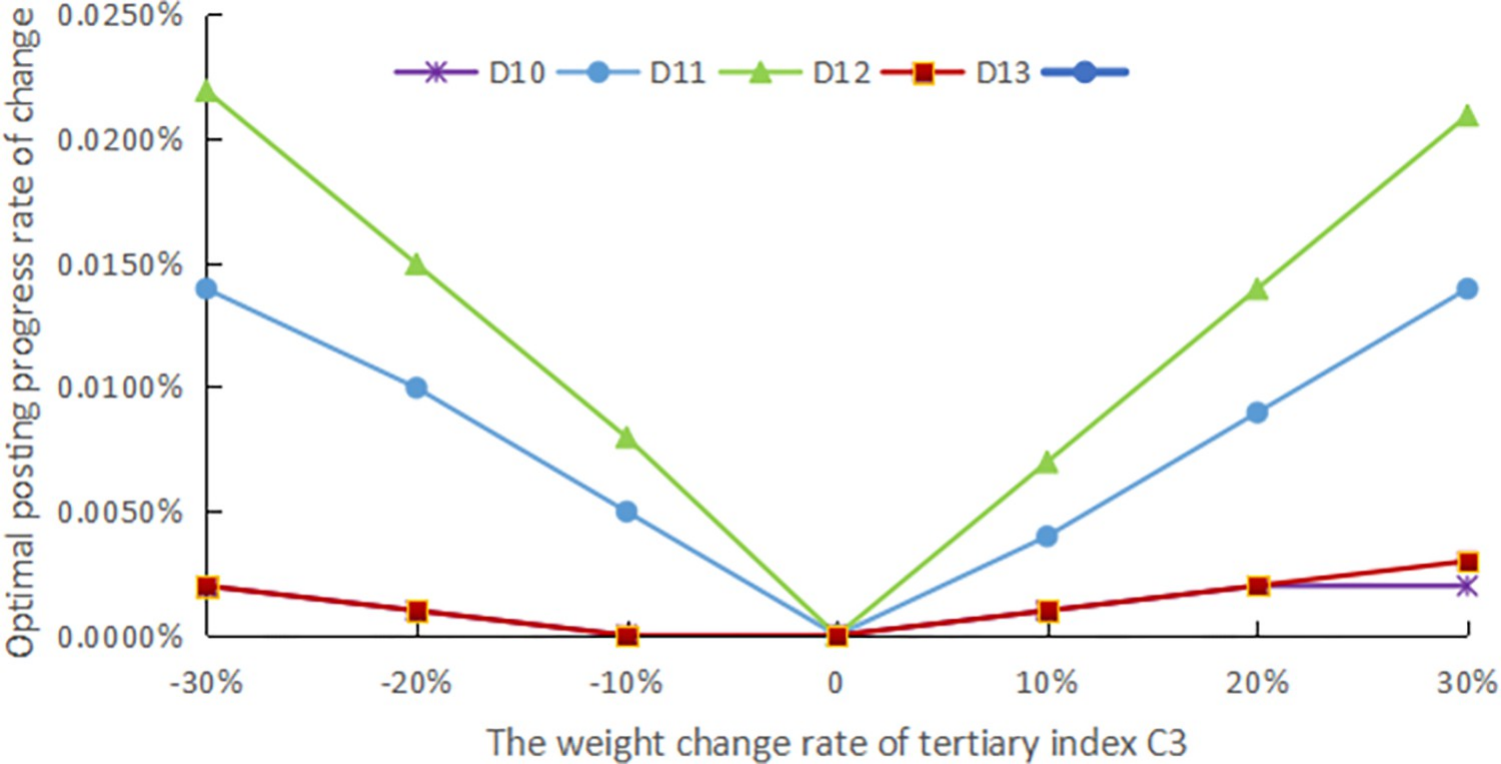
The weight change rate of tertiary index C3.

**Fig 8:**
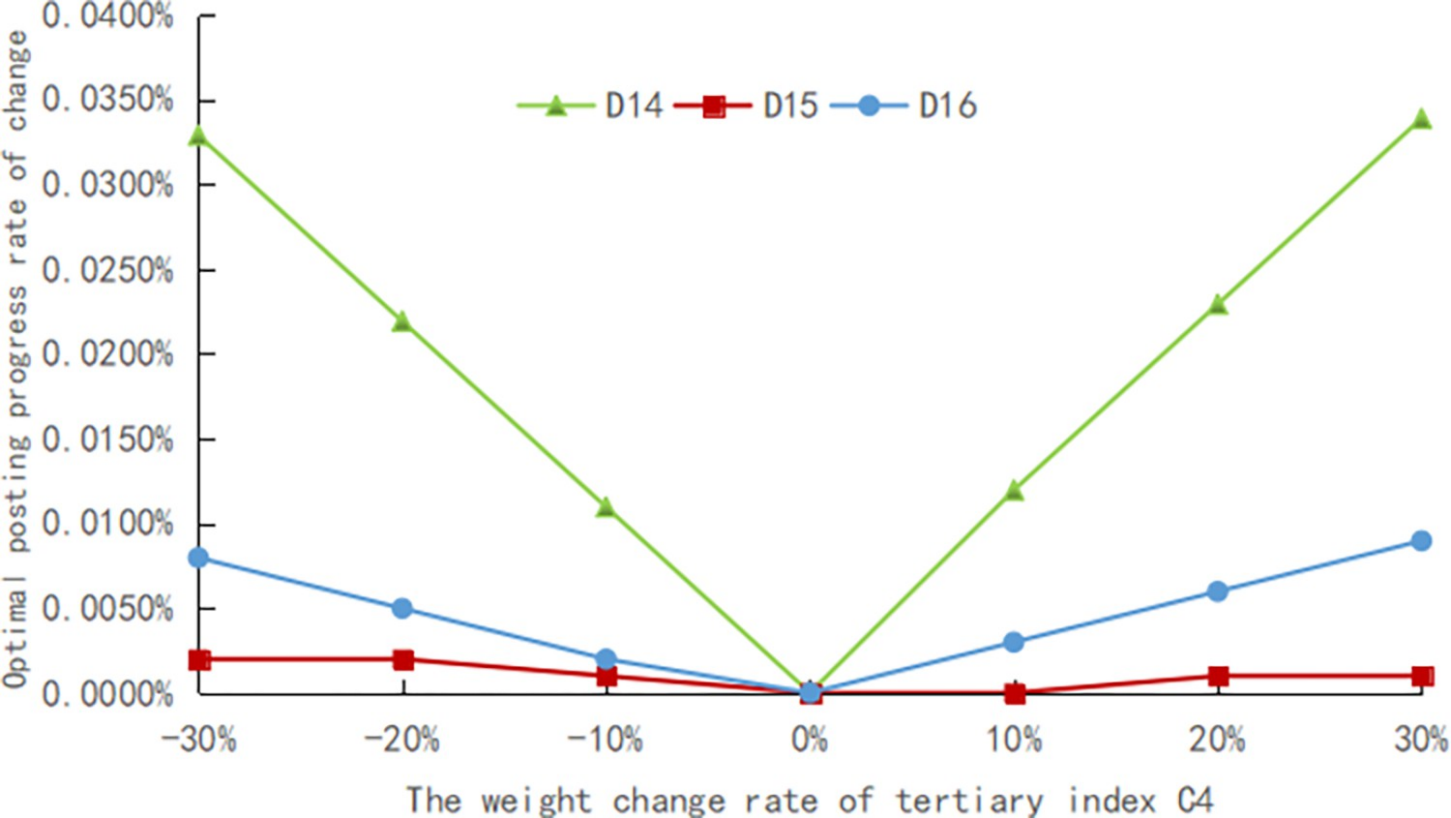
The weight change rate of tertiary index C4.

**Fig 9:**
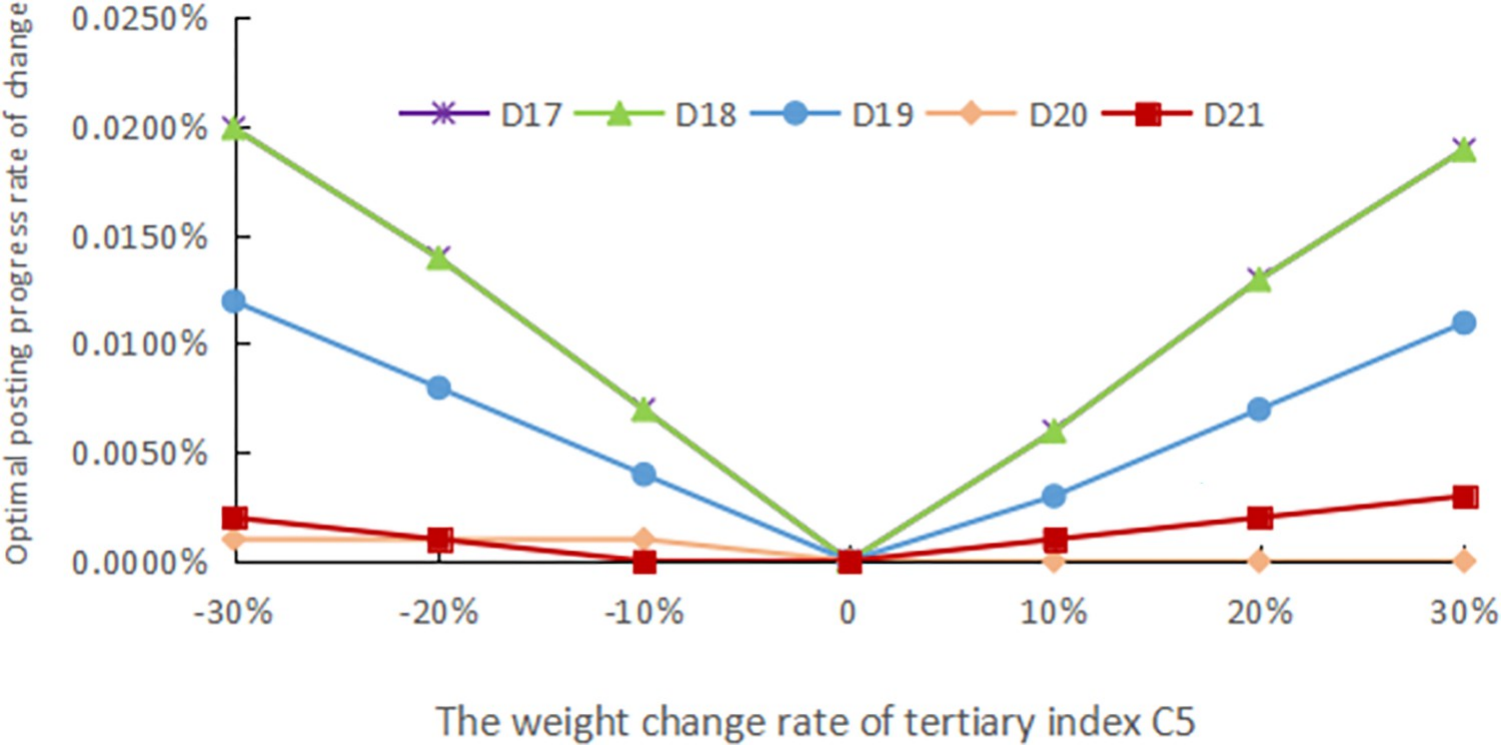
The weight change rate of tertiary index C5.

The evaluation method should have good robustness, and in order to confirm the robustness of the assessment procedure, sensitivity analysis is performed. In practical problems, deciding its assessment indicator system and the weights of each index with the help of expert experience is a simple and effective method, but the reliability of the assessment is unavoidably impacted by human subjectivity. As a result, in this article, the standard weights of each indicator are modified by ±10%, ±20%, and ±30%, respectively, to simulate the changes under different weight cases and compare the effects of these changes on the final evaluation results, as shown in Figs 5–9.

The weights of the three levels of indicators are modified separately. Finally, it still emerges that the advance of the digital countryside level in Jiangxi Province belongs to rank III, which is at a good development level. Figs 5 to 9 depict how the optimal posting progress (which is rank III) has changed in terms of absolute change rate with the change of the indicator weights. It is clear that each indicator’s absolute average change rate value has a different change rate value and is essentially symmetrically distributed with the weight change rate 0 at its center. This distribution also shows an approximately linear increase with increasing absolute weight change rate values. The absolute average change rate value is roughly the same when the absolute value of the weight change rate for a specific assessment factor is the same, meaning that it is similarly sensitive to the assessment outcomes. Meanwhile, it can be seen that D4 has the most enormous sensitivity to the weights, and the largest absolute mean rate of change value is 0.0329%, which is significantly less than the weight change rate size, demonstrating that assessment outcomes are somewhat steady. Therefore, the evaluation method has good robustness.

### 5.4 Comparative analysis

As a way to verify the rationality and accuracy of the proposed method, the AHP-entropy weighting approach and the coupling degree assessment model were applied to assess the advance of digital countryside in Jiangxi Province, and the results were good, which demonstrated the validity of the suggested approach.

In comparison to the AHP-entropy weighting method, the improved CRITIC modified G1 weighting method used in this paper retains the importance ranking of indicators and reflects the information size contained in the indicators, taking into account the mutual influence of indicators, and does not require consistency testing, which is simpler and more accurate in determining the weights of indicators. Compared with the coupling degree evaluation method, the topological element method used in this paper focuses more on the feasibility and optimization of the evaluation scheme, introducing the correlation degree function for quantification, and the calculation entirely takes into account the compatibility of the evaluation indicators, which is more operable, realistic and has more practical value.

## 6 Discussion and recommendations

The digital countryside’s construction accelerates rural areas’ sustainable development through digital empowerment. Digital rural construction is the strategic direction of rural revitalization and an essential path to realizing rural revitalization, injecting new momentum into rural revitalization. First of all, the construction of the digital countryside can transform traditional agriculture, realize intelligent management, and improve the efficiency and quality of agricultural production. It can also promote rural e-commerce, Internet entrepreneurship and innovation, and realize the industrialization of the rural digital economy to help the industry prosper and lead a prosperous life. Secondly, digitalization promotes the governance of the rural ecological environment and living environment, which can build a monitoring and management platform through digital technology, promote the treatment of domestic sewage and garbage, improve village appearance and other issues through information means, and help ecologically livable. In addition, digital governance can make farmers feel more secure and involved in rural governance. Digital rural government services can make business handling more convenient, rural operations are more open and transparent, medical, education and other resources more balanced distribution in urban and rural areas, and help rural civilization and effective governance. Therefore, the construction of the digital countryside can promote the overall goal of rural revitalization: "prosperous industry, livable ecology, civilized village style, effective governance, and prosperous life".

The construction of the digital countryside is a systematic project involving many stakeholders, such as the government, villagers and social organizations. The key to promoting the construction of the digital countryside is to promote the interaction and interest balance among these stakeholders [46]. The Chinese government attaches great importance to the construction of digital countryside. As the main body to promote the implementation, it can make relevant policies to involve many stakeholders to varying degrees, which is the critical lever for developing digital countryside construction. Social organizations, such as enterprises and research institutions, have advanced research and development capabilities and technologies, actively participating in the construction of digital villages, which is conducive to shaping a good image, increasing profits, and improving their comprehensive competitiveness. For villagers, the construction of digital villages is closely related to their lives and plays an irreplaceable role in the construction of digital villages. For example, in implementing energy community activities in Italy, particular emphasis is placed on citizens as the centre of this process, treating them as co-producers to solve problems rather than passive subjects and turning citizens into participants in the energy market [47]. Therefore, only by fully mobilizing the active initiative of stakeholders and building a collaborative governance pattern can the development of the digital countryside be promoted [48]. In view of the problems in the growth of the digital village, this essay provides the following suggestions.

(1) Promote the cultivation of digital talents.

The implementation of any national strategy is inseparable from professionals. For example, Greece’s major obstacle in adapting to climate change is the lack of human resources of public institutions [49]. The same is true of digital village construction. The implementation of the digital countryside strategy requires a large number of digital talents to support it. Since the digital countryside strategy has been proposed for a relatively short period, professional talent resources related to the digital countryside are relatively scarce. Therefore, it is necessary to launch a particular talent support plan around digital technology, take the lead in setting up specialized courses in local colleges and universities for artificial intelligence and the Internet of Things and other fields, encourage research institutions and social organizations such as e-commerce enterprises to explore joint educational modes, conduct qualification certification of relevant talents, and gradually select and train a group of people who have master information technology and rural development.

(2) Guarantee the source of funds for construction

As the main policy maker, the government has a prominent role in leading, coordinating and supervising the advance of the region under its jurisdiction. Policy and financial support from governments at all levels can effectively promote the progress of the region. Firstly, in the implementation of digital village development, governments at all levels should encourage grassroots organizations in villages to cooperate with various enterprises, guiding all sectors of society to participate in local digital village development. Secondly, each region should jointly fund and establish a regional digital village cooperation and development fund, specifically for cross-regional digital village infrastructure construction, inter-regional benefit compensation and multi-regional digital technology promotion. Through multiple parallel approaches, all parties are attracted to participate, thus ensuring the steady advance of digital villages.

(3) Improve the modernized governance system of the digital countryside

The United States develops digital agriculture through cross-border cooperation, aiming to give full play to its resources and achieve win-win results [50]. Japan has implemented an extensive subsidy policy for digital agriculture and has started with an intelligent transformation plan for agricultural infrastructure to realize the digital transformation of farms [51]. Although China’s digital village construction has made some progress, it is highly dependent on the government, mainly relying on government-led pilot demonstration projects. It is necessary to learn from the experience of digital rural development in other countries, make the best of it, and base it on local. By promoting the adhibition of digital techniques such as 5G, the Web of Things and big data in agricultural development, remote access to information are realized, realizing intelligent control of facilities, remote control of all kinds of Windows, nets, spray, drop and other equipment, and achieve convenient control, through mobile phones, PCS and other terminals to see, control and manage agricultural production. The introduction of these digital technologies into agricultural development has optimized the agricultural production system, which can improve the utilization efficiency of inputs such as fertilizers and the operation efficiency of agricultural machinery, reduce the carbon emission of crop systems, and promote the cost saving and efficiency increase of agriculture [52]. At the same time, actively explore new models such as "Internet + Party building", lead the "Internet + community" model to cover rural areas, share and analyze data resources for digital rural construction through a geographic information system, build a digital governance platform, make data information more open and transparent, and improve the rationality of rural governance.

(4) Accelerate the formulation of development plans

In promoting the energy community in Spain, the lack of clear and concise regulations and policies has hindered its development [53]. In the process of campus renovation in Greece, relevant policies have been formulated based on the principle of co-design to promote climate-resilient urban renewal [54]. With the in-depth practice of digital rural strategy, administrations at all levels must fully utilize the constructive guiding role in the growth of digital countryside. According to the requirements of the digital countryside advance planning, cities and counties are units to speed up the formulation of the top-level design of digital rural progress, and build a new development mechanism of unified planning system, common development model, and regional coordination and linkage. When the government formulates new development mechanisms and policies, it should communicate more with villagers and social organizations based on the principle of co-design so that they can also become designers of digital villages. This way, we can speed up efficiency, solve misunderstandings, develop better ideas, and jointly design a satisfied digital village.

## 7 Conclusion

Digital rural construction is not only the strategic direction of rural revitalization but also can alleviate the increasingly severe climate problems, which have attracted attention at home and abroad. For the purpose of encouraging the advance of the digital countryside, a complete and scientific assessment of the regional digital countryside growth level is crucial. To address this issue, this essay establishes a comprehensive evaluation system of digital countryside development based on the CRITIC-G1 empowerment method and the topological element evaluation method in the context of rural revitalization. Firstly, 21 key influencing factors were selected by the Delphi method and PCA method to establish a comprehensive assessment indicator system of digital rural advance. Then the weighting method of CRITIC-G1 is applied to determine the weight of assessment indexes. Finally, the advance level of digital countryside in the region is evaluated by using the topological element method. Taking Jiangxi Province as an example, this paper evaluates the development level of digital village in Jiangxi Province from the provincial level, and the results are as follows: Firstly, the development level of digital village in Jiangxi Province is good and is getting better and better. Secondly, from different aspects, the digitization of infrastructure, services, economy, and green production all belong to a good level, and the digitization of life development is excellent. Finally, the digitization of services, the economy, and green production is retreating. By evaluating the provincial level of digital rural development in Jiangxi Province, we can understand the overall and various aspects of digital rural development in Jiangxi Province. According to the actual situation, the government can conduct macro-control on the development of digital countryside in Jiangxi Province, give full play to the development advantages of digitization of infrastructure and life, and make up for the shortcomings of digitization of service, economy and green production, so as to promote the construction of digital countryside. For other regions, just like Jiangxi Province, the evaluation model proposed in this paper can be used to evaluate the development level of the digital countryside in this region, understand the problems existing in the development process of the digital countryside, and then formulate targeted measures to promote the construction of digital countryside. In addition, the evaluation model proposed in this paper can also be used to evaluate the development of digital villages at the municipal and county levels, to clearly understand the local development situation of digital villages at the micro level, and then promote the construction of digital villages pertinently. By evaluating the development level of digital villages in different regions at different levels, micro and macro regulations are carried out in parallel to promote the overall development of digital villages in China.

The United States has developed modern industry and solved the labour shortage problem through digital agriculture. Japan, which lacks natural resources, mainly uses cutting-edge ICT technologies to develop digital agriculture to improve land use efficiency. While empowering agriculture with digital technology, New Zealand and other Western European countries also pay attention to the integration of rural primary, secondary and tertiary industries. Although China’s digital village construction has achieved some results, it mainly relies on the government to promote it, and it has not yet formed a digital village model with perfect digital infrastructure, cross-industry cooperation and profit. As a policy maker, the government needs to actively involve many stakeholders to jointly draw a beautiful blueprint for the digital countryside, which can also increase the authority and credibility of the government and facilitate the implementation of policies [55]. Given the above problems, this paper puts forward relevant countermeasures and suggestions, hoping to develop the digital countryside further.

The study in this paper also has limitations and shortcomings. First of all, the evaluation system cannot be improved due to the lack of experience in developing and constructing digital countrysides. With the development of digital village construction, the evaluation index system can be further improved, and the identification method of the evaluation index can be innovated. Secondly, because the research area is in the countryside, the acquisition of some data becomes a complex problem, and the subsequent research should try to ensure the quantity and validity of data. In addition, the CRITIC-G1 empowerment method and the topological element method proposed in this study have proved the viability and dependability of the method based on sensitivity analysis and comparability analysis. Next, comparative studies based on other evaluation methods can be discussed. Finally, this paper takes Jiangxi Province as a case to evaluate the development of its digital countryside from the provincial level. The follow-up study can extend the evaluation scope to all regions of the country and study the inter-regional disparity in the development level of the digital countryside and its reasons.

## Supporting information

S1 Data Data on indicators for Jiangxi Province from 2016 to 2021. (PDF)
