# Supplementary material for: Comprehensive evaluation of digital village development in the context of rural revitalization: A case study from Jiangxi Province of China
Source: PLoS One. 2024 May 16;19(5):e0303847. doi: 10.1371/journal.pone.0303847 (PMC11098496; doi:10.1371/journal.pone.0303847)
Supplement: S1 Data — (PDF) [file pone.0303847.s001.pdf]

Data on indicators for Jiangxi Province from 2016 to 2021						
Indicators	2016	2017	2018	2019	2020	2021
D1	3359287.00	3402721.00	3449893.00	3501529.00	3510103.00	352314.00
D2	136899.00	137771.00	137395.00	184404.00	185785.00	186161.00
D3	70.00	75.00	82.40	83.00	84.30	85.80
D4	244.00	309.30	415.30	460.90	518.50	556.80
D5	894.00	1067.00	1215.00	1425.00	1678.00	1896.00
D6	30394.00	29734.00	28309.00	28088.00	27440.00	27189.00
D7	180.10	175.70	167.80	142.30	146.90	142.60
D8	1844.10	1870.00	1844.10	1888.90	2078.00	2074.30
D9	17046.00	17033.00	17004.00	17005.00	16979.00	16994.00
D10	0.1961	0.2689	0.3307	0.4062	0.4852	0.5639
D11	223.76	267.17	296.23	319.13	340.61	364.56
D12	12138.00	13242.00	14460.00	15796.00	16981.00	18684.00
D13	89872.00	90373.00	90190.00	93489.00	90281.00	89568.00
D14	241.19	249.52	274.55	276.83	269.18	276.40
D15	98.50	98.40	98.80	98.90	99.40	99.40
D16	22.24	24.59	23.67	25.83	27.59	24.90
D17	2201.62	2309.60	2381.97	2470.66	2591.05	2695.35
D18	4044.00	4306.00	4647.00	4355.00	4523.00	4682.00
D19	2036.83	2039.42	2032.02	2036.14	2038.49	2038.50
D20	141.97	134.97	123.20	115.57	108.81	108.59
D21	5674.90	5787.30	5917.85	6070.46	6195.89	6333.42
